# Experiences of elder abuse: a qualitative study among victims in Sweden

**DOI:** 10.1186/s12877-022-02933-8

**Published:** 2022-03-28

**Authors:** Mikael Ludvigsson, Nicolina Wiklund, Katarina Swahnberg, Johanna Simmons

**Affiliations:** 1 Department of Acute Internal Medicine and Geriatrics and Department of Health, Medicine and Caring Sciences, Linköping University, Linköping, Sweden; 2 Department of Psychiatry and Department of Biomedical and Clinical Sciences, Linköping University, Linköping, Sweden; 3 Department of Health and Caring Sciences, Faculty of Health and Life Sciences, Linnaeus University, Kalmar, Sweden

**Keywords:** Aged, Neglect, Mistreatment, Ageism, Violence

## Abstract

**Background:**

Elder abuse is underreported and undertreated. Methods for prevention and intervention are being developed, but the knowledge guiding such measures is often insufficiently based on the victims’ own voices due to a paucity of studies. The aim of this study was therefore to explore experiences of elder abuse among the victims themselves.

**Methods:**

Consecutive inpatients ≥ 65 years of age at a hospital clinic in Sweden were invited to participate, and 24 victims of elder abuse were identified. Semi-structured qualitative interviews were conducted, and transcripts were analyzed using qualitative content analysis.

**Results:**

The analysis generated four themes that together give a comprehensive picture of elder abuse from the participants’ subjective perspectives. The participants’ experiences of abuse were similar to previous third-party descriptions of elder abuse and to descriptions of abuse among younger adults, but certain aspects were substantially different. Vulnerability due to aging and diseases led to dependance on others and reduced autonomy. Rich descriptions were conveyed of neglect, psychological abuse, and other types of abuse in the contexts of both care services and family relations.

**Conclusions:**

Elder abuse is often associated with an individual vulnerability mix of the aging body, illnesses, and help dependence in connection with dysfunctional surroundings. As individual differences of vulnerability, exposure to violence, and associated consequences were so clear, this implies that components of prevention and intervention should be individually tailored to match the needs and preferences of older victims.

**Supplementary Information:**

The online version contains supplementary material available at 10.1186/s12877-022-02933-8.

## Background

Abuse of older adults is recognized as a pervasive and serious problem in society. Prevalence estimates have ranged from 10% upwards in cognitively intact persons from North and South America, with large variations between different countries and subcategories of the population [1–3]. Elder abuse is defined by the World Health Organization (WHO) as “a single or repeated act or lack of appropriate action, occurring within any relationship where there is an expectation of trust, which causes harm or distress to an older person”. It includes five different types of abuse: physical abuse, psychological abuse, sexual abuse, economic abuse, and neglect [4]. Elder abuse is associated with various adverse health outcomes including psychosocial distress, morbidity, and mortality [1]. Exposure to more than one type of abuse or by more than one perpetrator is called poly-victimization, and this common condition is generally associated with even worse health outcomes than single exposure to abuse [5, 6]. It is also increasingly acknowledged that elder abuse is associated with previous experiences of violence in childhood and adulthood, motivating a life-course perspective in research on elder abuse [7, 8].

**Fig. 1:**
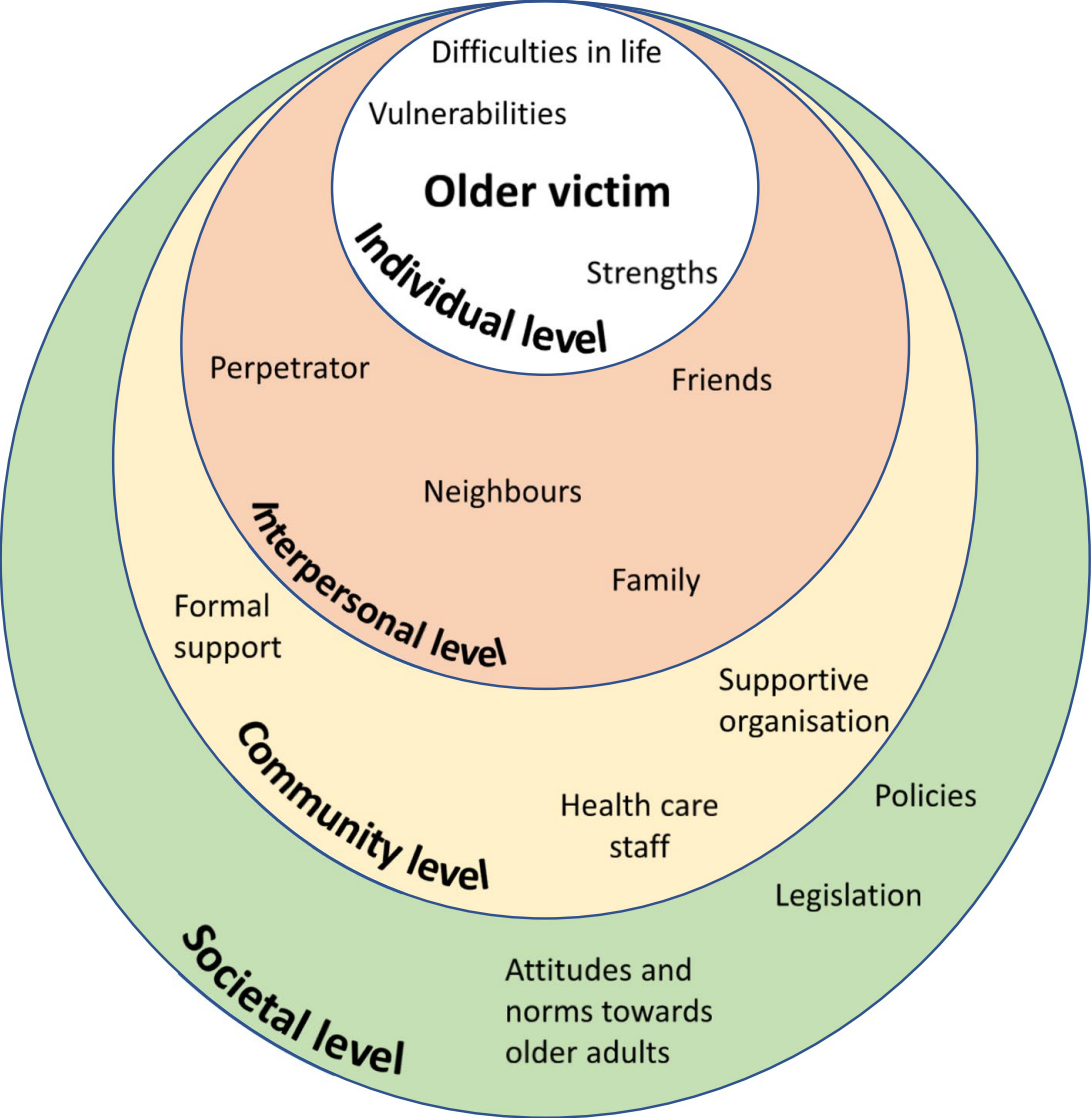
The socioecological model inspired by Bronfenbrenner [9] and Heise [14] as a mean to understand the complexity of elder abuse

The causes and mechanisms of elder abuse are important to understand, to prevent its occurrence more effectively in society. The socio-ecological model (Fig. 1) of abuse describes how abuse can be understood as a complex interplay between risk factors on different social levels (individual, relationship, community, or societal level) for the victim [5, 9]. By analyzing and handling abuse with help of this model, the circumstances of abuse are concretized which facilitates practical interventions. However, experiences of elder abuse differ between professionals, other surrounding persons, and the older adults themselves [10, 11], and the varying conceptions and definitions used have consequences for the types and forms of interventions planned. If supportive resources are not adapted to the victims’ conceptualizations of elder abuse or to their perceived needs, the resources risk being ineffective [1, 12, 13]. Thus, the voices of the victims themselves are important to truly understand their associated needs as well as the causes and mechanisms of elder abuse, in order to develop more effective interventions.

Furthermore, qualitative studies have been proposed to better understand conceptual and cultural variations of elder abuse [1]. Some qualitative studies on abuse of older adults have been undertaken within a theoretical framework of intimate partner violence (IPV; [15, 16]), but this framework differs from the framework of elder abuse for example by underestimating the categories of abused men, neglect, and abuse by personnel in healthcare or long-term care [17–19]. Abuse in healthcare and long-term care are particularly relevant for a comprehensive picture of elder abuse as increasing proportions of the population encounter such institutions due to increasing age, frailty, and social dependence [2, 20].

Within the framework of elder abuse, several qualitative studies have asked professionals or other third parties about elder abuse [11, 21, 22], but only few have asked the victims themselves [21, 23, 24]. However, these few previous studies do not offer a sufficiently comprehensive picture of the matter which is why we conducted the present study.

## Methods

### Aim

The aim of this study was to explore experiences of elder abuse among the victims themselves. By asking the victims directly, our understanding of elder abuse can hopefully deepen and this in turn is essential for adequate prevention and intervention.

### Design, setting and sample

Semi structured qualitative interviews were conducted and analyzed using content analysis. The sample was 24 participants from the larger REAGERA (Responding to Elder Abuse in GERiAtric care) project, which included developing and validating the screening instrument REAGERA-S for detecting elder abuse in healthcare [25]. Consecutive older adults ≥ 65 years of age admitted to a hospital clinic for both acute geriatric and acute medical patients were eligible for inclusion. The consecutive sampling was chosen in the pursuit of naturalistic openness, and this sampling was expected to lead to a wider range of abuse (including mild forms of abuse), compared to alternative purposeful sampling strategies. A parallel goal of gathering information-rich data was reached through a relatively large number of participants. Exclusion criteria were insufficient somatic, cognitive, or linguistic capacity to answer the screening instrument either independently or with the help of healthcare personnel. Patients at the clinic were mostly admitted from the emergency department, and the mean duration of stay for patients over 65 years was 10 days at the acute geriatric ward and 4 days at the acute medical ward during the study period. The setting is described in greater detail elsewhere [25]. Between January and June 2018, 306 potential participants were asked to participate by nurses on the ward. The screening instrument was completed by 191 participants, of which 135 were interviewed. Of these 135 participants, 24 had been victims of elder abuse and all their 24 recorded interviews were included for this qualitative study. Descriptive data about the 24 included participants are presented in Table 1. Typically for the setting of the hospital clinic, the mean age was rather high, as were the number of medications and the degree of social dependence for managing activities of daily living—compared to an average patient in health care.

**Table 1:** Background characteristics of included participants for the interviews, *n* = 24

	**n (%)/ mean[sd]**	**Item non-response**
Social and demographic variables		
Age, mean [sd]	81 [8.0]	1
Female sex, n (%)	15 (62.5)	-
Higher educational level, > 9 years of education, n (%)	13 (54.2)	-
Assisted living, n (%)	3 (12.5)	-
Living alone, n (%)	13 (54.2)	-
Someone to talk to, n (%)	20 (95.2)	3
Functional and medical variables		
Help with Instrumental Activities of daily living, n (%)	16 (66.7)	-
Help with Basic Activities of daily living, n (%)	6 (27.3)	2
Need of wheelchair or stroller, n (%)	15 (68.2)	2
Help with medications, n (%)	6 (26.1)	1
Number of medications, mean [sd]	15 [5.8]	6

### Procedures

Before the interview, a nurse on the ward distributed a questionnaire to potential participants including the screening instrument REAGERA-S [25], as well as information about voluntary participation and informed consent. The screening instrument included nine questions about different kinds of abuse (e.g." Has anyone attempted to control you, limit your contact with others, or decide what you may or may not do?”;”Have you been subjected to any form of physical violence, for example being shoved, pinched, held down, hit or kicked?”), and one question about associated suffering. The instrument in total is available elsewhere [25]. No precise definition of elder abuse was presented for the participants before the interviews. Rather the information preceding the interviews included rather vague descriptions of elder abuse (e.g. “to be subjected to negative actions”) to prevent steering the participants’ thoughts or stories for the data collection. Later that same day or the following day, a qualitative interview was conducted in a private room. The interview was semi-structured using a prepared interview guide (see Supplement 1), with four main topics to cover (experiences of abuse, associated thoughts and feelings, effects of the abuse, and support after the abuse). The informants’ experiences of abuse are presented in this study, while their experiences of coping with abuse and their desired support are presented in a separate paper.

For the interviews, we used open-ended questions such as “Can you tell me some more about what you were exposed to?” and “What are your feelings when you think about this today?”. Probing and supplementary questions were also asked. The interviews were audio recorded and transcribed verbatim. The length of the interviews varied between 12 and 97 min. Field notes were written during or after the interviews. Just after each formal interview, the previously completed questionnaire was quickly checked for severe depression or suicidal risk. In two cases, this check – together with the interview – resulted in a referral to an appropriate care unit for support connected to being abused. The individual’s responses from the REAGERA-S were used at a later stage when classifying cases of elder abuse after the interview, described in more detail elsewhere [25]. All participants received both oral and written information about support services to contact in case of need. In addition to checking the participants’ psychological wellbeing and perceptions of participation in the interviews, additional follow-ups were carried out by phone by the researchers about 1–2 weeks after the interviews. All participants gave written informed consent at the time of participation. A potential ethical problem of the consent process was the principal vulnerability of the participant in the hospital care setting. The interviewers (three of the researchers: JS, NW and ML) usually work as physicians but were not involved in the formal care of the participants, and this was communicated to the patients orally and through a civilian clothing. By signaling thus that the interviewers were separate from the formal health care personnel, elements of vulnerability and potential dependency of the participant was prevented in the participation. Also, security and rapport were built in the meeting through active listening and validation. The study was conducted in accordance with the principles of the Declaration of Helsinki and approved by the Regional Ethics Review Board in Linköping, Sweden (2017/181–31; 2017/564–32).

### Analysis

Transcripts were analyzed using qualitative content analysis, based on Graneheim and Lundman [26] and a hermeneutic standpoint with an intermediate level of abstraction and interpretation [27]. For the purpose of exploring individual experiences, the qualitative content analysis was considered an appropriate method with a focus on subject, context and variation of the data [27]. The process of analysis involved the following steps: 1) repeated preliminary readings of unique interviews to obtain a sense of the whole; 2) dividing the text into units of meaning; 3) giving codes to condensed meaning units; 4) abstraction within and between interviews by aggregating codes into tentative subcategories/categories (manifest content), and subthemes/themes (latent interpretive content); 5) discussion and revision of tentative codes, subthemes/themes, and subcategories into more definitive ones. The analysis included both a search for convergent patterns and a mirror analytical strategy to investigate divergence (consideration of data that did not fit the dominant patterns) [28].

Six interviews were coded separately and were then discussed together by all the authors. For the remaining interviews, the coding and development of tentative subcategories and themes were carried out by two of the authors (ML and JS; steps 1–4). The tentative themes/subthemes were then discussed and revised (step 5) by all the authors together. This validation within the research group aimed to strengthen the research design, not by identical statements or consensus but as a form of reflexivity through contesting and supplementing each other’s readings [29]. The QSR International’s NVivo 12 software program was used as a means for sorting and managing data during the process.

## Results

**Fig. 2:**
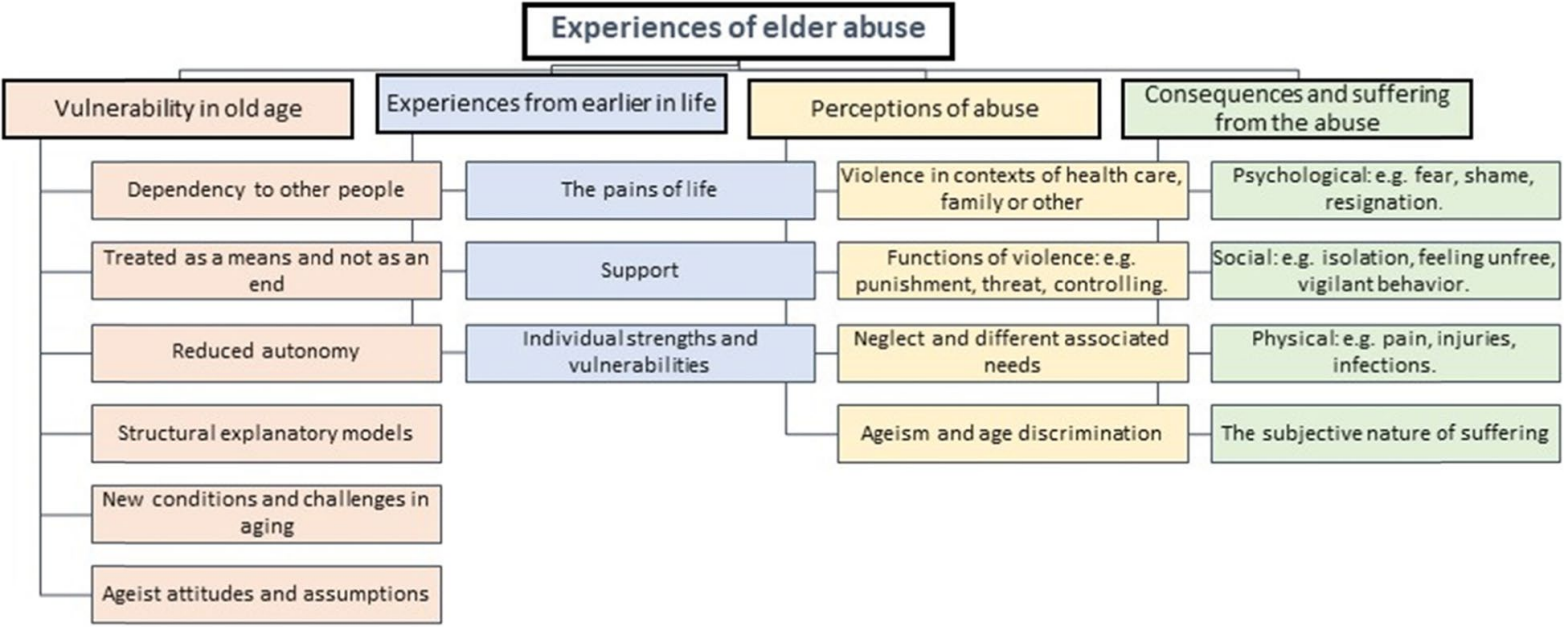
Coding tree as an overview of the themes, subcategories, and subthemes of the qualitative content analysis

The qualitative analysis generated four themes which are presented in detail below: vulnerability in old age; experiences from earlier in life; perceptions of abuse; consequences and suffering from the abuse. An overview of the themes subthemes and subcategories is depicted in Fig. 2.

### Vulnerability in old age

The participants described their life situations as contexts for the adverse events they had been subjected to, and these descriptions expressed a general pattern of vulnerability. This vulnerability largely consisted of different kinds of dependence on other people: social, physical, and medical dependence. Social dependence sometimes reflected efforts to avoid loneliness, conflicts, sorrow, or other adversities for the family members.

Physical dependence could be the need to get a ride to visit friends, or a need for assistance with putting on socks due to reduced mobility, while medical dependence could be a need for assistance with injection treatment. The participants’ vulnerability was due to the natural consequences of normal aging, including a lack of energy or reduced mobility, or the consequences of illness, with reduced capacity for activities and participation. It was also a result of social relationships that had evolved over the course of a long lifetime. When participants asked for help or received help from those around them, they consequently had reduced defense against or increased vulnerability to abuse.

“Well, I’m not a happy person any longer, I’m hardly allowed to laugh, because he doesn’t like that really. […] And I also don’t get outdoors like I did before. Then I could take the bus downtown and go shopping and do whatever I wanted. Now he’s behind my wheelchair, checking me all the time, and that’s not fun.” (Woman, ID 9, 71 years).

The participants often expressed a desire to overcome their dependence, either by managing on their own or by finding alternative helpers. Thus, the dependency was often related to a specific perpetrator, but also in general related to any potential helper. However, a lack of energy or failing capacities during old age often resulted in dependence remaining. As a part of their vulnerability, the participants also expressed that it was hard to defend themselves when exposed to abusive situations:

“… if you have employees who behave a little badly to you, that’s different [that’s one thing]… But if you encounter resistance in healthcare, that’s another story.” (Man, ID 19, 85 years).

How the participants related to their vulnerability or their dependence varied, although a common approach was the desire not to bother their helpers (relatives or personnel).

“[There were] times when they [the care personnel] didn’t come. They have… they had a shortage of staff, and when some of them got sick they skipped [visiting some patients], and I was probably the one they cared about the least, as I was the most alert of us.” (Man, ID 1, 85 years).

Reduced autonomy was also described as an aspect or a consequence of dependence on help, whereby the older adults were not allowed to decide, or could not decide, about their life situation. Their autonomy was sometimes reduced by the limited willingness or ability of those around them to meet their needs. On other occasions, their autonomy was reduced by their physical or social impairments. For example, they were sometimes not allowed to decide where to live, or which activities to engage in.

“And they’re talking about putting me there again [in the nursing home], and I don’t want that, but what the hell can I do [about it]?” (Man, ID 1, 85 years).

The participants also expressed their perceptions of limited autonomy when they were treated like objects rather than individuals, or when the personnel did not show any interest or engagement in their personal needs, desires, or personality. For instance, all residents at the nursing home were invited – or sometimes rather forced – to participate in certain specific activities, due to the mistaken ageist notion that all older adults enjoy the same sort of activities. Thus, the older adults perceived reduced autonomy when grudgingly participating in bingo competitions.

The participants also conveyed their theories about why their dependence became so problematic, and these theories were often about specific members of staff being perceived as unfriendly or incompetent. Other theories related to how structural deficiencies of society – or of healthcare, or of certain organizations – contributed to a general lack of humanity among the older adults’ potential helpers. An example of such a perceived structural deficiency is when financial savings made by an organization are allowed to trump care quality or staff competence in healthcare. Accordingly, a recurrently suggested intervention to prevent elder abuse would be to educate the care staff:

Interviewer: “How would it be possible to … [prevent age-discriminatory care by the assisted living]?

Participant: “By educating the care staff, of course…. So to [that they would] understand that an older adult has a background whatever that may be./…/. Perhaps education [for them], to understand the individual, so to say”. (Woman, ID 3, 84 years).

### Experiences from earlier in life

In addition to the above descriptions of vulnerability during old age, the participants also spoke about their earlier lives, including time of adversity and joy. Several older participants described that, during old age and beforehand, they could receive strength or support from a friend or a partner, from family members, or by participating in an organization. These surrounding supporting elements helped to create security and meaningfulness, despite the adversities of life.

“She [my wife] was valuable to me… in all kinds of ways. And I have always encountered love through church, and these things have been very valuable to me.” (Man, ID 19, 85 years).

Some participants highlighted their activities or professional experiences that had provided support in life, while others highlighted important insights or mental attitudes that had helped to form their identities, their inner strengths, or their sense of meaning in life.

“When I grew up […] I had to do as I was told. And with this attitude I have managed.” (Man, ID 6, 76 years)

Alongside the participants’ stories about positive experiences and support throughout life, they also conveyed rich stories about difficulties and adversities in life. These stories were often about being a victim of violence during childhood, for example being subjected to school bullying or experiencing different types of violence in the family.

“I was five years old when I saw my father threaten her [my mother] with a loaded rifle, then she was wedged into a corner and he stood in the middle of the floor. […] Then my childhood ended, that day.” (Woman, ID 8, 73 years).

The participants told their stories about being subjected to violence in the past with such passion and emphasis that it became clear during the interviews how violence – even many years ago – could have just as strong an impact on health as recent events of victimization.

### Perceptions of abuse

In the interviews, the participants described all five types of abuse. Patterns of neglect and psychological violence were most prominent in their stories, while economic, physical, and sexual violence were generally less prominent.

*Neglect* occurred in relation to different helpers that the participants were dependent on, and the neglect was related to a variety of needs. Hygiene needs were neglected when the participants had limited access to help with showering, cleaning or washing services, or clothing. Insufficient assistance with buying food or medication was described in association with staff shortages at the care organizations, which could prevent the older adults from initiating treatment prescribed by a doctor.

Neglected medical needs could involve sloppy or incompetent wound dressing, or when staff often forgot to administer medications. Several stories related to how care staff dismissed the older adults’ medical needs or symptoms, on the incorrect ageist assumption that the symptoms were signs of normal aging. The following quotation was interpreted as an example of age discrimination, and at the same time neglect of medical needs when a woman was refused a regular treatment regime. It was unclear whether the neglect was intentional or not.

“I was in France last year. I went down a mountain, skiing, it was slippery. [I] was going down and then got stuck in a fence, and so I twisted my knee. […] [I waited two days to seek healthcare until I came home from the journey.] And then they tell me ‘Well, because of your age you’ll have to wait for six months [to receive care]’, oh my god, and ‘You’ll have to do physiotherapy and attend to the osteoarthritis school’.” (Woman, ID 18, 69 years).

Examples of social needs being neglected varied in nature. This could involve older adults being frequently forgotten, after staff had said “I’ll be right back” in response to a request for help. Alternatively, social needs could be neglected when older residents at a nursing home were forced to attend social activities that were not in line with the individual’s specific preferences or abilities. A lack of staff continuity could mean that the participants were deprived of steady relationships with other people. In such ways, the participants expressed a lack of a meaningful existence, secondary to the social neglect.

“And the nursing home was so… well, it was so boring, damn it! It was as if a lot of… I don’t know what to call it… zombies [demented people] went around. They didn’t talk. That [living situation] wasn’t stimulating, either for me or for them.” (Man, ID 1, 85 years).

*Psychological abuse* was often connected to neglect and occurred in healthcare as well as in nursing homes and in family environments. The psychological abuse was often perceived as a means by which to control or manipulate the participant’s actions. This control could be about small matters, like the choice of which food to eat, but it could also be about more important matters like whether or not to request home service. Sometimes the abuser used aggressive speech if the participant did not live up to the abuser’s expectations or demands.

“I’ve talked to him about it [getting home service], but he doesn’t want that, because he thinks it’s too expensive. But I just feel I don’t have energy to do anything. And he says [to me]: ‘You’re so damned lazy.’” (Woman, ID 9, 71 years).

Control was sometimes exerted verbally, but often involved more subtle non-verbal expressions, such as constant surveillance in daily activities, or expressing a non-verbal tone of disapproval if the participant met friends. In one case, a woman had even been prevented from seeing her mother on her death bed:

“So when my mother was dying, they called me [from her town] and told me to come as there was not much time left. […] I’ll come right away I said, I’ll get on the first flight. And then my husband told me I couldn’t go as it was the weekend, and that I should wait until Monday. […] I wanted to say goodbye [to her] anyway, I wanted to be with her. But I never got there in time, they called me on Monday morning and said she was gone. […] And I hate this.” (Woman, ID 7, 66 years).

The controlling behavior often turned into direct threats against the participant from a child or a partner. These threats could be related to physical violence or not being allowed to see their grandchildren anymore. Psychological abuse also occurred in care environments, although the expressions were generally less explicit. In healthcare, just as in family environments, the abuse was perceived as an attempt to control the older participant’s behavior. Often the intent of the staff seemed to be well-meaning, but the expression was perceived aggressive or otherwise negative by the participant. One example was the following situation, where the participant had just completed a cardiac exercise test at the hospital:

“… I had cycled very fast, I was in severe pain and I was lying on the bed. […] and then she [the member of staff] would, at the physician’s request, spray nitro medication under my tongue, which she did and said to me: ‘Shut your mouth and swallow’, but I couldn’t because I was just in cramp… so she says again ‘Shut your mouth and swallow!’ but I still couldn’t do it, and then she turns away and says ‘Well then, forget that shit!’.” (Woman, ID 8, 73 years).

The descriptions of *economic abuse* that emerged during the interviews were many and rich in character. Sometimes the perception of economic abuse was not primarily associated with the lost financial value, but rather with the feeling of deception after a theft within a relationship of trust, or the feeling of sorrow when the lost item had great sentimental value.

“I felt terrible [when the jewelry was stolen by service staff], and after that I have never again… asked [them] for help. […] Yes, I think a lot about the jewelry being gone… it was a necklace that I had inherited from my mother, and a bracelet…” (Woman, ID 23, 73 years).

Stories about *physical abuse* during aging were few, but there were more examples of this from earlier in life. Examples of physical violence in old age including a robbery necessitating hospital care, being pushed by an official during a home visit, physical violence from a fellow passenger during transportation services, and one participant being hit by hospital staff.

“I’ve been hit on the head with a pillow. Just because I was cranky, she [the nurse] said. And I didn’t like that… And I said: ‘Now you get out of here, because you shouldn’t be working with people.’ […] [I] think it is frightening when you have to go to a care facility to receive care, and then you get hit! I don’t think it is acceptable.” (Man, ID 6, 76 years).

There were several stories about *sexual abuse* from earlier in life, but only few from old age. In one case the participant had been recurrently raped within the marriage, but the raping had ended some years before the age of 65. In another case, sexual abuse in contact with healthcare staff had obviously occurred during old age.

“Once, there was a physician that made some – it sounds weird now that I’m 84, I think I was ten years younger then – he really made sexual invitations [to me]. Yes, I think it sounds weird, but I felt very awkward.” (Woman, ID 3, 84 years).

The participants’ stories of sexual abuse expressed clearly feelings of shame and disgust.

### Consequences and suffering from the abuse

The abuse that the participants had been exposed to led to various consequences. Psychological consequences included uncomfortable or painful feelings or thoughts that tormented the participant long after the abuse. For example, this could include nervousness, depression, disappointment, or guilt on the part of the abuser or the victim. The intensity of these uncomfortable feelings and thoughts varied over time, with a common gradual decrease as time, ordinary life, or support measures had helped to sooth the remaining discomfort. However, even a long time after the abuse had ended, the painful feelings and thoughts could be brought back by events or conversations, so that the intensity became strong again. Even if the interviews themselves evoked such painful feelings, the participants generally perceived the interviews as positive.

“… Because I sense this, how can somebody just do that? It’s [the painful experience]… Yes, it’s inside me. I try to get rid of it when it comes, but it isn’t so easy, sometimes it just comes and yes, it’s just there.” (Woman, ID 10, 67 years).

The participants described feelings of inferiority or uselessness, even though they tried to convince themselves that such feelings or thoughts were not truthful. Feelings of nervousness and fear increased again when experiencing new threats of abuse, for example when facing a new need for hospital care after previous negative experiences of abuse in healthcare.

“I hate being admitted [to hospital] like this, you don’t know which department you will be admitted to or which staff you will meet. […] You’re always prepared for the worst. You never know who you will meet when you’re admitted… Of course, I’m always on my guard… against a punch or such things.” (Man, ID 6, 76 years).

The fear of being robbed again made the participants vigilant and distrustful toward staff, strangers, and authorities. Lasting harm from abuse could include aches due to internal tension. Although the participant conveyed that the physical symptoms were caused by the abuse or medical errors, such causal relationships or physical consequences sometimes seemed uncertain for the researchers.

Social effects of the abuse could include loneliness, avoiding going outdoors due to fear of violence, or social isolation caused by reduced self-confidence or an abuser limiting their personal freedom. Social effects could also include a reluctance to accept care service due to fear, even though the older adult needed such services. Regardless of whether the abuse was ongoing or in the past, the suffering could be so intense that the person had lost the will to live or even planned to take their own life.

“I wouldn’t be alive if I didn’t have them [the children]. Then [without the children] I’d have been gone [dead] a long time ago. Then, I wouldn’t be alive. I don’t like life that much.” (Woman, ID 7, 66 years).

The participants commonly expressed feelings of abandonment and lack of control, in association with the abuse and their situation. By contrast, a few participants instead conveyed how they continued to defend their autonomy and strove to keep control of the situation through different strategies, despite their limited physical condition due to old age.

### Discussion and implications

This aim of this study was to explore experiences of elder abuse among the victims themselves, as their own descriptions can help us to better understand how to develop prevention and interventions against elder abuse. The qualitative analysis resulted in four different themes (vulnerability in old age; experiences from earlier in life; perceptions of abuse; and consequences and suffering from the abuse), which describe different aspects of abuse from the participants’ subjective perspectives. In all, many of the participants’ perceptions of abuse were similar to previous descriptions by third party of elder abuse [11, 21, 22]. Some aspects of the descriptions of elder abuse in this study were also similar to previous descriptions of abuse among younger adults, but other aspects were substantially different [30–32], as discussed below.

### Vulnerability in old age and experiences from earlier in life

Vulnerability to abuse during old age was described as different sorts of dependence on other people, and a lack of autonomy. Due to the effects of normal aging or accumulated diseases, the participants had limited mobility and an increasing need for care in everyday life, which meant dependence on care and vulnerability to abuse from others.

When the participants were exposed to abuse, their ability to defend themselves was also low for the same reasons. In general, this contributed to a submissive attitude toward the helper, together with inner reactions of anger, sorrow, and resignation. These descriptions of vulnerability have similarities with descriptions of vulnerability and powerlessness among younger adult victims of abuse in healthcare and other settings [32, 33]. At the same time, the context of the aging body is characteristically different for the older adult, with decreasing capabilities and increasing dependence on care. The participants’ vulnerabilities were very varying and unique to each individual in terms of aging, morbidity, and life experiences.

The descriptions of vulnerability in old age were similar to those recounted by Y Mysyuk, RG Westendorp and J Lindenberg [23]. Dependence was described as a reciprocal process between the abuser and the victim in Mysyuk et al., something that was not spontaneously conveyed from the participants of this study. Nor did we identify the pattern described in Mysyuk et al., whereby increased weakness or dependence would provoke more violence.

The participants’ stories about previous stages of their life contributed to comprehensive individual pictures of how specific abuse in old age had had impact on their health. It was particularly evident that abuse in the past could have a great impact on health in old age, for example when psychological abuse in childhood had additive or synergistic effects on the perception of elder abuse. This is in line with previous literature on poly-victimization, and underlines that understanding elder abuse presupposes considering previous victimization as well as personality and the victims’ experiences of support, attachment styles, and challenges in life [5, 8, 34]. According to the socio-ecological model of abuse (Fig. 1), vulnerability can occur on all levels of an individual’s life, although previous experiences of life mainly correspond to the individual and interpersonal levels for the older adult [5, 9]. Previous life experiences are important not only for understanding the individual’s unique vulnerability to abuse, but also for considering the victim’s individual strengths and resources when designing interventions and the prevention of elder abuse [35]. Hence, our results agree well with previous findings that a life-course perspective is essential when trying to understand the causes and consequences of elder abuse [6–8]. However, our findings also underline that abuse occurs in a context, and factors on all levels of the socioecological model influence the experience of abuse, e.g., ageist attitudes and dysfunctional care organizations described further on. By paying attention to and validating the older adult’s own life story, staff can indirectly contribute to interventions at community level in accordance with the socio-ecological model, as this level includes how the victim is treated by organizations [19].

### Different kinds of elder abuse, ageism, and perceived causes of elder abuse

Neglect was a common kind of abuse in this study, and there were rich descriptions of this from healthcare settings and long-term care institutions. Not only were physical and medical needs neglected – so, too, were social needs, with consequent intense feelings of abandonment and lack of control among the participants. These descriptions were partly similar to those found in previous studies [36], although the examples of neglect in this study were often modest in character, meaning potentially mild physical adverse effects in the short term. Nevertheless, also modest shortcomings with hygiene or cleaning could have serious or even life-threatening consequences, as they meant an increased risk of serious wound infections. Ageist attitudes were obvious in different types of abuse, and especially in the descriptions of neglect, in which for example all older adults were treated like objects in a routine way without respect for their individual characters, needs, or preferences.

The psychological abuse occurred in both family and care environments and seemed to correspond to the abusers’ attempts to control the participants’ behaviors. In care environments, the abuse could be a way for staff to control behaviors in line with specific care routines or comfortable forms of work for the staff. The descriptions of psychological abuse in this study were similar to previous descriptions of psychological abuse in younger victims in healthcare and in younger persons in other environments [30, 33, 37].

The participants often added their own personal explanations for the abuse. In addition to descriptions of vulnerability and self-blame, common explanations included individual staff members being unfriendly, care organizations being structurally dysfunctional (with a lack of competence and resources), general greed at all levels of society, and discriminatory (ageist) attitudes and actions leading to neglect. Similar explanations have been described in previous studies, with ageism probably corresponding to all levels of the socio-ecological model [11, 23, 38, 39]. Some people would perhaps think that structural deficiencies are not relevant to abuse, but the very definition of elder abuse by WHO clarifies that also “lack of appropriate action” in a dysfunctional environment can constitute elder abuse [4].

A general issue from the analysis of the interviews was whether the WHO definition of elder abuse is too narrow since it limits elder abuse to relations of trust. In several examples there was no identified relation of trust in a reported situation, but rather a “situation of trust” in which the abusive action would best be described as an example of elder abuse. For example, when an older adult is exposed to abuse during transportation services, there would be a situation of trust regardless of whether there are any relations of trust. The older adult would typically be vulnerable in this situation due to the physical limitations of ageing. With a narrow interpretation of the WHO definition, this abuse would dysfunctionally not be classified as elder abuse, although the theoretical framework of elder abuse would fit for an adequate understanding and prevention of the same abuse [40].

### Consequences of the abuse

The participants described consequences of abuse in a way that resembled how consequences of trauma have been previously described among both older and younger adults [3, 30, 41]. Whereas patterns of psychological consequences (with negative thoughts and feelings of shame and fear) were rather like descriptions from previous studies among younger adults, the behavioral consequences were different and related to various social and physical preconditions among the older adults compared to younger adults. Social isolation and loneliness were natural consequences of limited mobility in normal aging or disease, and when abuse also contributed to these limitations the sense of isolation grew particularly strong. When participants chose not to receive home-care services because of the fear of recurrent abuse – despite their needs for assistance – the limiting consequences of abuse were particularly evident. There were also examples from the interviews of how neglect could have serious potential physical consequences, as many of the older adults were less physically able to withstand medical mistreatment.

### Implications for the prevention of and intervention into elder abuse

Our findings have several implications for the prevention of and intervention into elder abuse. In terms of the socio-ecological model, preventive measures at community (including hospital level) and societal levels could be to ensure a minimum standard (for example by using legislation or economic incentives) for the care of older adults. According to the participants' voices, higher minimum standards of staff competence and resources would be likely to reduce the tendencies toward neglect, psychological abuse, or other kinds of abuse. Vulnerability and abuse could according to the participants also be prevented through education to care staff about different aspects of elder abuse and about aging. Such educational measures were suggested to promote person-centredness and prevent ageist attitudes, as these attitudes seem to contribute to both the vulnerability and elder abuse [39]. In addition, support units are also needed to offer individual assistance to victims of elder abuse as the negative consequences are substantial. According to a bifocal ecological approach, the assistance should not only be directed to the victim for an effective prevention but also to the perpetrator [42]. However, an important principle should be to adapt the preventive measures to the individual, as both vulnerability and abuse perceptions vary significantly according to the individual’s unique biopsychosocial conditions and experiences from earlier life. This also underlines the need for a life-course perspective on elder abuse [34, 43].

### Limitations

In order to minimize bias introduced by the researchers’ preconceptions and instead promote reflexivity, four researchers with different backgrounds have cooperated in the study. Three of the authors work as physicians within geriatrics and psychiatry, while the fourth author works with research, mostly outside hospital environments.

The fact that the sample was selected from inpatient care could be regarded as a disadvantage, as some older adults might have had too little energy to participate actively in interviews while suffering from an acute illness with associated physical exhaustion. On the other hand, the decision to recruit participants from inpatient care meant certain advantages, for example offering the participants a secure context for the interviews while their home or other environments might have been less secure, or more easily controlled by an abuser.

The results are likely to be transferable to older adults in Sweden but should be transferred with caution to countries with other cultures or societal structures.

## Conclusions

This is one of few studies to date in which qualitative interviews have been used to explore experiences of elder abuse among the victims themselves. Their stories had similarities with both previous third-party descriptions of elder abuse and previous descriptions of abuse among younger adults. There were also substantial differences, with the consequence that elder abuse needs to be understood and managed by partly different means compared to abuse among younger adults. Vulnerability to elder abuse is often associated with an individual mix of the aging body, illnesses, and a dependence on secondary help. A life-course perspective considering experiences from the individual’s past would be beneficial when designing support for older victims, as such experiences are important to the degree of suffering and disability that the victim develops in relation to elder abuse. Prevention ought to include individually tailored help or support to reduce vulnerability, specific education, and ensuring an acceptable minimum standard of care for older adults in general [14].

## Supplementary Information

**Additional file1.** **Supplement 1.**INTERVIEW GUIDE (for two parallel studies).

## Data Availability

The datasets generated and analyzed during the current study are not publicly available and are not available from the corresponding author on request due to reasons concerning participant privacy and confidentiality.
